# Dexamethasone Creates a Suppressive Microenvironment and Promotes *Aspergillus fumigatus* Invasion in a Human 3D Epithelial/Immune Respiratory Model

**DOI:** 10.3390/jof7030221

**Published:** 2021-03-18

**Authors:** Maureen K. Luvanda, Wilfried Posch, Asma Noureen, Eliott Lafon, Viktoria Zaderer, Cornelia Lass-Flörl, Doris Wilflingseder

**Affiliations:** Institute of Hygiene and Medical Microbiology, Medical University of Innsbruck, 6020 Innsbruck, Austria; Maureen.Luvanda@i-med.ac.at (M.K.L.); wilfried.posch@i-med.ac.at (W.P.); asma.noureen@i-med.ac.at (A.N.); eliott.lafon@i-med.ac.at (E.L.); viktoria.zaderer@i-med.ac.at (V.Z.); cornelia.lass-floerl@i-med.ac.at (C.L.-F.)

**Keywords:** dexamethasone, *Aspergillus fumigatus*, 3D lung model, macrophages

## Abstract

Lung immunity and susceptibility to infections is subject to interactions between the epithelial layer and immune cells residing in the pulmonary space. *Aspergillus (A.) fumigatus*, the most prevalent pathogenic fungus, affects both upper and lower respiratory tracts of immunocompromised hosts. Several reports implicate corticosteroids as a major risk factor due to their anti-inflammatory and immunosuppressive effects, which are exacerbated by long-term treatment regimens. Here we demonstrate for the first time the influence of dexamethasone when it comes to germination and hyphae formation of *A. fumigatus* in the presence of macrophages within a highly differentiated air–liquid interphase (ALI) epithelial/immune lung model. We illustrate suppressed mucus production within the highly differentiated 3D respiratory model as well as significantly decreased cilia beat frequencies by dexamethasone treatment. This goes along with corticosteroid-mediated macrophage M2 polarization within the epithelial/immune microenvironment. Therefore, we here showed that corticosteroids promote enhanced fungal growth and invasion *A. fumigatus* by creating a suppressive environment affecting both epithelial as well as immune cells.

## 1. Introduction

Almost every therapeutic agent is currently screened in animal models before clinical trials and translation [1]. However, animal models have considerably different physiological and genetic compositions compared to humans [2]. The generation of more human-like in vitro models incorporating complex parameters, such as primary cells and extensive vasculature, is imperative during preliminary drug screening studies so as to avoid situations where animal experimental outcomes do not translate reliably, such as in the TGN1412 trial disaster of 2006 [3]. Additionally, with regards to fungal infections, several animal models, such as *Galleria mellonella, Drosophila melanogaster,* and even mice, have been found more susceptible to some melanin mutants of *Aspergillus (A.) fumigatus,* deeming them unsuitable models to replicate the human condition [4]. An assortment of cells makes up the pseudostratified epithelium; these include basal cells, ciliated, club cells, and goblet cells [5,6]. Basal stem cells continually differentiate into surface cells. Ciliated cells actively eliminate debris and unwanted particles from the pulmonary system in conjunction with club as well as goblet cells which secret mucus, surfactants, and antimicrobial fluids leading to direct impediment of microbial development [7]. These cells collectively participate in the first-line defense against pulmonary invasion of pathogens in association with immune cells, such as macrophages [8]. This effect generally affects several respiratory pathogens, which mainly attempt to colonize, invade or disseminate within the host via inhalation [9]. Since the epithelial lining covers the majority of the human airway [10], it generally determines the outcome of respiratory diseases. 

*Aspergillus fumigatus* is a challenging respiratory pathogen in immunocompromised individuals. Especially in those receiving long-term immunosuppressive treatments [11,12,13]. It causes various pulmonary diseases, which are grouped depending on the extent of invasion and region of colonization. These diseases include invasive *Aspergillosis* (IA), an extremely serious condition with a mortality rate up to 95% in untreated cases and around 40% in treated ones. Through Glucocorticoid Receptor-mediated signaling, steroids regulate cellular functions, metabolism, differentiation, survival, proliferation, and general immune responses [14,15]. These endogenous substances are cholesterol-derived hormones produced by the adrenal gland [16], but synthetic versions have been developed and are commonly applied whenever potent immune regulation is required [17]. Here, we study the impact of dexamethasone on *A. fumigatus* virulence in an environment simulating an in vivo immunocompromised pulmonary system. To do so, we utilized a pre-established 3D lung model [18] to recapitulate the morphological and biochemical features of a fungus-infected respiratory tract containing lung epithelial and first-line immune cells allowing us to investigate the cross-talk between the different cells of the airway in the presence of the common immunosuppressing therapeutic, dexamethasone.

## 2. Materials and Methods

### 2.1. Dexamethasone Preparation and Treatment

Dexamethasone powder (Sigma, St. Louis, MO, USA #D4902) was diluted in absolute Ethanol (Carl Roth, Karlsruhe, Germany #D65.1) to a final stock concentration of 20 mg/mL as previously described [15]. It was then stored at −20 °C until needed. Dilutions in the relevant media were done according to experimental purposes shortly before performing the experiments. In its diluted form, it was stored in the dark at 4 °C for only one week, and fresh dilutions were prepared at the end of the period to prevent degradation. Samples were treated with dexamethasone concentrations ranging from 0.1 to 500 µg/mL *v/v* or vehicle alone. Dex or vehicle (EtOH) were applied in experiments for 7 days to simulate long-term treatment.

### 2.2. Isolation of Monocytes and Macrophage Differentiation

Due to the limited availability of pulmonary macrophages [19], donor monocytes were derived from normal healthy donor whole blood buffy coats using anti-human CD14 Magnetic Particles (BD ImagTM, Franklin Lakes, NJ #557769, USA) and cell separation magnets (BD ImagTM, Franklin Lakes, NJ #552311, USA). Purity was confirmed using flow cytometry as previously described [15]. Polarization was accomplished using 50 ng/mL rhGM-CSF (#572905, Biolegend, San Diego, CA, USA) for 7 days to obtain M1 (GM-MDMs, corresponds to GM-CSF-generated monocyte-derived macrophages [MDMs])-like macrophages, respectively [15].

### 2.3. Respiratory MODEL 

Primary normal human bronchial epithelial (NHBE) and small airway epithelial (SAE) cells were obtained from Lonza (Basel, Switzerland) and cultured as previously described [18]. NHBE and SAE included in all studies were of Passage 2 or lower in consideration of their primary nature to eliminate any chances of mutational confounding factors that may affect the experiments and interfere with the epithelial tight junction integrity. In brief, they were seeded into a 75 cm^2^ Tissue culture flask (TPP/Biomedica, Divischgasse, Vienna, Austria, #30076) and cultured at 37 °C in a 5% CO_2_ humidified incubator and left to grow to ~80% confluence for 5 days. Cells were then harvested and transferred onto collagen-based 0.33 cm^2^ (0.4 µm) transparent porous matrices (polyester membrane inserts) at a density of 1 × 105 cells/Transwell (Stemcell Technologies, VN, Canada #380023/#380024). They were then sustained in differentiation media according to the manufacturer’s instructions.

### 2.4. TEER

During cell differentiation, the trans epithelial electrical resistance (TEER) of cultured cells was analyzed using STX2 chopstick electrodes and the EVOM volt–ohm-meter (Word Precision Instruments, Sarasota, FL, USA) to confirm the tight and healthy structure of the airway epithelium, as previously described [18].

### 2.5. Macrophage–NHBE Coculture 

Once the system was confirmed to be stable and fully differentiated, cocultures of differentiated NHBE cells at air–liquid interphase (ALI) with macrophages were generated. The immune cells were added to the apical side, simulating the luminal surface of the epithelium [20]. Before addition, macrophages were harvested using a cell scraper (Greiner, Kremsmüster, Austria #541070), seeded onto cell cultures of respiratory epithelial cells under static conditions, and observed for pathogen-specific effects on their immune parameters.

### 2.6. Chemotaxis and Transmigration Assays

To confirm the phenotypic identity of the cells following treatment, 1 × 10^5^ MDMs were loaded on top of the aforementioned Transwell Permeable Inserts (BD Biosciences, Franklin Lakes, NJ #353493, USA), which contained a pre-grown and differentiated, pseudostratified epithelial layer at ALI. They were then transferred into wells that contained media plus different concentrations of dexamethasone, and 50 ng/mL rhGM-CSF was applied as a positive chemoattractant to the lower chamber. Incubation was then carried out for 24 h at 37 °C, after which the membranes were cut out, stained, fixed, and mounted on slides (ThermoFisher Scientific, Waltham, MA #WT4301672, USA) and coverslips (VWR, Radnor, PA #631-0657) with Mowiol (Carl Roth, Karlsruhe, Germany #0713.2) applied as the essential mounting medium. Immune cell migration characteristics were analyzed using computational 3D imaging (Perkin Elmer, Operetta Confocal Microscope, Waltham, MA, USA). The protocol was a loose adaption from [21]. 

### 2.7. Aspergillus (A.) fumigatus Culture 

The dsRed-expressing isolate of *A. fumigatus* strain number AF293 was utilized in this study. The organism was grown on *Aspergillus* Complete Media (ACM) for 5 days at 35 °C as previously described [15,18]. Conidia were then harvested and suspensions filtered using a sterile 40 um pore size filter (BD Falcon, Franklin Lakes, NJ #352340, USA).

### 2.8. Multi-Color Flow Cytometry

Flow cytometry was performed to analyze surface marker and intracellular protein expression as previously described [18]. In summary, *A. fumigatus* infected and uninfected cells were centrifuged and suspended in flow cytometry wash buffer (Dulbecco´s Phosphate-buffered saline [DPBS] #D8537 +0.5% bovine serum albumin [BSA] #05473) (both obtained from Sigma, St. Louis, MO, USA) + 5 Mm ethylenediaminetetraacetic acid [EDTA] (#1128309, ThermoFisher Scientific, Waltham, MA, USA) + 0.1% Sodium Azide (Sigma, St. Louis, MO, USA #S2002). They were washed once with DPBS at (1000 rpm, 5 min, +4 °C) and stained with fluorescently labeled monoclonal antibodies (mAbs). Labeling was done using anti-human Dectin–2-Phycoerythrin [PE] (R&D Systems, Minneapolis, MN, USA #FAB31148), anti-human CD206 fluorescein [FITC] (#551135), anti-human E-Cadherin Alexa Fluor^®^ 647 (#560062), both from BD Pharmingen, Franklin Lakes, NJ, USA, anti-Pan Cytokeratin AE1/AE3 (#53–9003-82 and Mitotracker^®^ Orange CM–H2TMRos (#M7511), both from Invitrogen, Carlsbad, CA, USA. Since Cytokeratin and E-cadherin are intracellular markers, permeabilization of NHBE cells was carried out using the 1× Perm/Wash solution (Biolegend San Diego, CA, USA #421002) before staining in the same solution. Data acquisition was carried out by FACS Verse flow cytometer (BD Biosciences, Franklin Lakes, NJ, USA). For each analysis, 10,000 events were collected, and live/dead analyses were applied to gate-out dead cells. FACS DIVA Software (BD Biosciences, Franklin Lakes, NJ, USA) was utilized for analysis. Data were depicted as mean fluorescence intensity (MFI). 

### 2.9. A. fumigatus Infection of the 3D Respiratory Model

Conidia were seeded into the 3D lung model cocultures at an MOI (Multiplicity of Infection) of 1 to immune cells (0.2 × 10^6^). The challenge was carried out for 24 h at 37 °C in a 5% CO_2_ humidified incubator. Cultures of conidia without any macrophages served as controls. 

### 2.10. NHBE Membrane Immunostaining, Mounting and HCS (High Content Screening)

To evaluate the exact phenotypic modifications elicited due to dexamethasone treatment of the 3D lung model, high-content, high-throughput confocal evaluation was applied (Operetta CLS, Perkin Elmer, Waltham, MA, USA). Once experimentation was complete, NHBE membranes were mechanically sliced from the Transwells using a surgical blade. They were then stained using the reagents mentioned in Table 1, dependent on experiment protocol, then washed and mounted as previously described [18]. Z-stacks were performed to ensure different parameters were captured and >3 fields were measured per slide. Live cell imaging was also carried out using the non-confocal mode to capture cilia movements and mucociliary clearance. To ensure statistical significance, experiments were carried out in triplicate, and the results were analyzed using Harmony SoftwareTM (Perkin Elmer, Waltham, MA, USA).

### 2.11. ELISA for Cytokine Assessment

Cytokines secreted within the 3D respiratory model following coculture with macrophages were analyzed using enzyme-linked immunosorbent assay (ELISA). These included interleukin (IL)-1β (#437004), IL-6 (#437004), IL-8 (#431504), and IL-10 (#4306049). Cell-free culture supernatants using the commercially available ELISA MAX™ Deluxe Set (Biolegend, San Diego, CA, USA) specific for each cytokine of interest were measured according to the manufacturer’s instructions. Cytokine quantification was determined as previously described [15]. 

### 2.12. Statistical Analysis

Statistical analysis was performed using GraphPad Prism Software, Version 8.0. (GraphPad Software Inc., San Diego, CA, USA). Average values were then computed for analysis, and unless otherwise stated, all data were reported as Mean ± SD of >3 independent experiments. Differences between groups were examined using one-way ANOVA and Dunnett’s posttest. The Student’s *t*-test was used for comparisons against a single control group. Probability values < 0.05 were considered significant. 

## 3. Results

### 3.1. Dexamethasone Does Not Disrupt Epithelial Integrity

**Figure 1:**
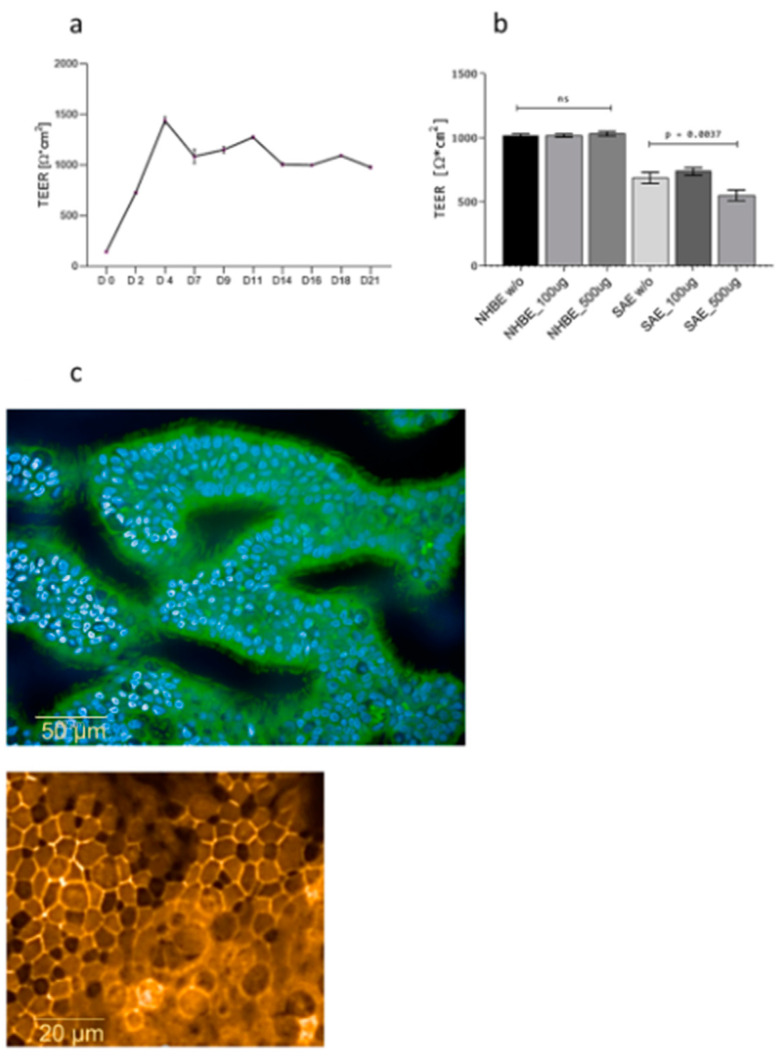
(**a**–**c**) Dexamethasone does not have an impact on epithelial integrity. (**a**) Transepithelial electrical resistance (TEER) values before dexamethasone treatment. Differentiation of the pseudostratified layer was carried out for 21 days under air–liquid interphase (ALI). Values were retained at >400 Ohms.cm^2^ from Day 2 with fluctuations then stabilization at Day 14 (1089.33 ± 46.36 Ω* cm^2^), indicating intact membrane integrity. (**b**) TEER values following 7-day dexamethasone treatment. Differentiation of the pseudostratified layer was carried out for 21 days under ALI. After differentiation was complete, primary normal human bronchial epithelial NHBE cells, as well as small airway epithelial (SAE) cells, were treated with 100 µg mL^−1^ and 500 µg mL^−1^ dexamethasone for 7 days, and epithelial integrity was analyzed using TEER. No differences in the epithelial integrity of NHBE cells were observed. However, SAE was significantly impacted at the higher concentration used. (**c**) Phenotypic analyses of dexamethasone-treated tissues using confocal microscopy. Representative images depict an intact ciliary structure (upper panel) and tight junctions (lower panel) in 3D epithelia treated with 500 µg/mL dexamethasone. Images were taken using a 40× water objective, staining was done using Hoechst 33,342 (blue) for nuclei, WGA-Alexa Fluor^®^ 488 (green) for ciliated structures, and Occludin592 (orange) for tight junctions. Scale bar as indicated.

Cell viability of 3D respiratory tissue models was analyzed following long-term treatment with dexamethasone to detect early indicators of toxicity. No differences in cell survival were observed following treatment with various concentrations of dexamethasone. During the differentiation period of 21 days in air–liquid interphase (ALI), TEER values within the 3D respiratory model were analyzed to confirm the integrity of tight junctions (Figure 1a). TEER values were corrected for resistance and surface area of Transwell (TW) filters as previously described [18], and they remained stable after differentiation with values of 1089.33 ± 46.36 Ω* cm^2^. These analyses indicated the integrity of the membrane was reliable before experimental applications. Following dexamethasone treatment, there was no significant decrease in TEER values in 3D differentiated NHBE cells (Figure 1b), while SAE cells from the lower respiratory tract seemed to be more sensitive to basolateral dexamethasone addition (Figure 1b). Since tight junction establishment is a requirement for a properly functional barrier [22], phenotypic analysis of tight junctions between adjacent cells was further performed using confocal microscopy (Figure 1c, Supplementary Video S1). These analyses revealed that upon long-term, basolateral treatment, even with the highest dexamethasone concentration (500 µg/mL), ciliary structures (Figure 1c, upper panel, ciliated cells green, nuclei blue), as well as tight junctions, were not affected (Figure 1c, upper panel, orange; Supplementary Video S1). Therefore, we here illustrated that dexamethasone does not disrupt epithelial integrity. 

### 3.2. Dexamethasone Exerts an Impact on Mucus Production

**Figure 2:**
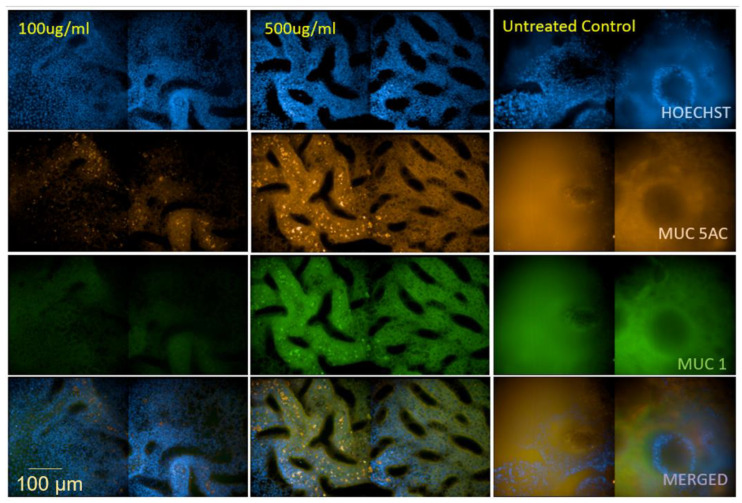
Dexamethasone reduces mucus production on airway epithelial cells. The respiratory 3D model was treated with dexamethasone (100 µg mL^−1^ and 500 µg mL^−1^) for 7 days. Following staining for MUC1-Alexa488 (green) and MUC5AC-Alexa555 (orange), phenotypic differences were assessed using confocal microscopy. Representative images of NHBE cells in ALI grown on collagen showing differences in MUC5AC and MUC1 expression, which was downregulated following dexamethasone treatment. Membranes were counterstained using Hoechst 33342 to highlight nuclei (blue). The experiment was independently repeated three times, and multiple fields were imaged.

To characterize whether dexamethasone has an effect on mucus production, we evaluated the abundance of mucins MUC5AC and MUC1 in primary differentiated epithelia. We here found considerably reduced expression of both MUC5AC (Figure 2, orange) and MUC1 (Figure 2, green) after adding 100 µg ml^−1^ and 500 µg ml^−1^ dexamethasone to the basolateral medium for 7 days. Downmodulation of MUC5AC and MUC1 was higher at lower dexamethasone concentrations. The reduced mucus production in dexamethasone-treated cultures was already visible analyzing brightfield images of untreated and treated Transwell membranes (Figure S1). These analyses reveal that long-term dexamethasone suppresses mucus production on airway epithelial cells.

### 3.3. Dexamethasone Reduces Ciliary Beat Frequency within the 3D Lung Model Independent of A. fumigatus Infection

Live cell imaging of untreated (Supplementary Video S2a and S3a) and dexamethasone-treated (Supplementary Video S2b and S3b,c) human 3D respiratory tissues, differentiated for ~80 days in ALI, was performed, and differences in ciliary beat frequency (CBF) were observed using the 5× (Supplementary Video S2a,b) and 20× air objectives (Supplementary Video S3a–c). Analyses of time-series images to create the movies were done using the Harmony software (Perkin Elmer). CBF was automatically tracked at intervals for 15–30 min. Supplementary Video S2 depicts an overview of brightfield movies from control and 500 µg/mL dex-treated cells, while in Supplementary Video S3, close-ups of an overlay of brightfield and digital phase contrast movies from control tissues (Supplementary Video S3a) and tissue models incubated for 7 days using 100 µg/mL (Supplementary Video S3b) or 500 µg/mL (Supplementary Video S3c) Dex are depicted. These analyses were in addition performed following infection with a dsRed-strain of *A. fumigatus* (Supplementary Video S4a–c). In addition, these analyses revealed strongly reduced CBF between control tissues (Supplementary Video S4a1,2) and tissues treated with 100 µg/mL (Supplementary Video S4b1,2) or 500 µg/mL (Supplementary Video S4c1,2). Supplementary Videos S4a1–c1 depict an overlay of brightfield (cells) and red (*A. fumigatus*, orange) channels, while Supplementary Videos S4a2–c2 illustrate the red channel only (*A. fumigatus*, orange). A clear reduction in CBF was observed in dexamethasone-incubated, highly differentiated epithelial tissue models, too, if they were infected with *A. fumigatus*.

### 3.4. Dexamethasone Induces an Immunosuppressed Microenvironment Characterized by CD163 Expression and Obstructed Macrophage Trans-Epithelial Migration

**Figure 3:**
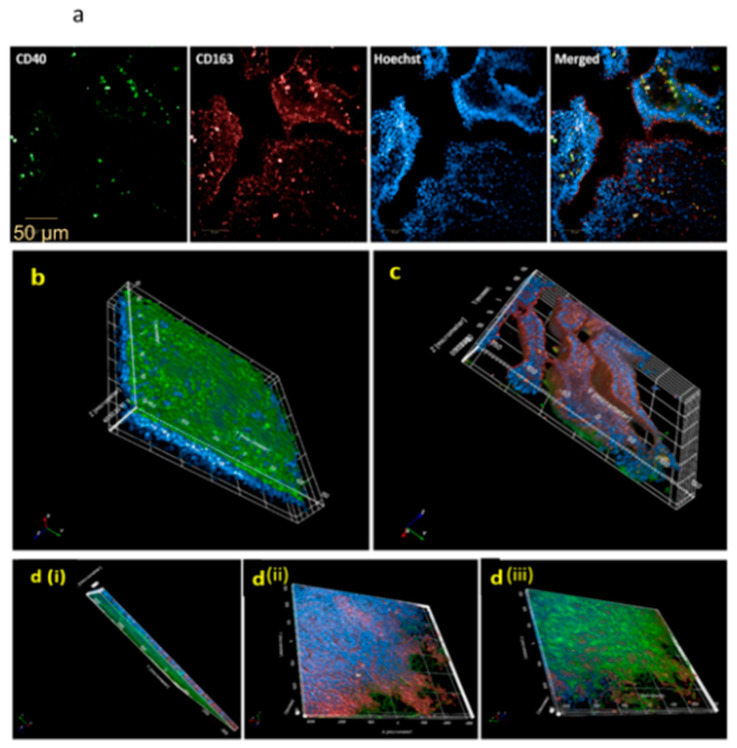
(**a**) CD163 expression 3D viewing within the 3D lung model. Within the 3D lung model treated with dexamethasone (100 µg mL^−1^), macrophages expressed both CD40 (Alexa Fluor^®^ 488, green) and CD163 (Alexa Fluor^®^ 647, red), indicating the establishment of an immunosuppressed microenvironment. Scale bar_ 50 µm. The experiment was repeated three times independently. (**b**–**d**) Macrophage chemotactic migration of M1 macrophages (CD40^+^) is mediated within the 3D microenvironment, while CD163^+^ macrophages remain on the apical side. Following 24-h stimulation using GM-CSF as a chemoattractant, (**b**) GM-MDMs (CD40^+^ green) migrate to the bottom of the membrane (basolateral distribution), whereas (**c**) Dexamethasone-induced-MDMs (CD163^+^ red) stay localized at the top of the membrane (apically lodged). **d**(**i**,**ii**) Imaging at different angles, X is the lateral axis and Y depicts direction. At least three independent experiments were performed, and at least three different areas of the membrane were imaged. 20XW Objective, N.A. 1.0. Hoechst 33,342 was applied to label nuclei.

We recently illustrated in macrophage cultures that dexamethasone initiated a skewing of M1 into M2 macrophages [15]. Therefore, following the coculture of MDMs and NHBE cells within the dexamethasone model, the expression of the M2-associated CD163 surface marker was evaluated. The membranes were, therefore, stained using the CD40 Alexa Fluor^®^ 488 (green) to visualize M1 macrophages, CD163 Alexa Fluor^®^ 647 (red) for M2 macrophages, and Hoechst 33,342 (blue) for nuclei. As seen in Figure 3a and Supplementary Figure S2, there was a clear expression of both surface markers, CD40 and CD163, indicating that untreated M1-MDMs are distinct from Dex-MDMs within the model, implying a difference in the microenvironments. Moreover, in a process known as chemotaxis, healthy, well-functioning immune cells migrate in response to external stimulation [23]. Therefore, we next examined the impact of dexamethasone on macrophage migration towards a recombinant human GM-CSF (50 ng/mL) gradient. We illustrated by 3D confocal imaging that GM-MDMs (CD40^+^) successfully migrated from the apical to the basolateral side within 24 h, whereas, as expected, dexamethasone (Dex)-MDMs (CD163^+^/CD40^−^) were retained on the apical side despite chemotactic stimulation as seen in Figure 3b–d (also see Supplementary Figure S3). These results not only provided evidence of dexamethasone’s impact on MDM movement, but they also verified the identity of Dex-MDMs to the alternative phenotype, an observation that we had recently made in macrophage monocultures [15].

### 3.5. Dexamethasone Treatment Skews Epithelial/Immune Lung Tissues to an Anti-Inflammatory Pattern Independent on A. fumigatus Infection

**Figure 4:**
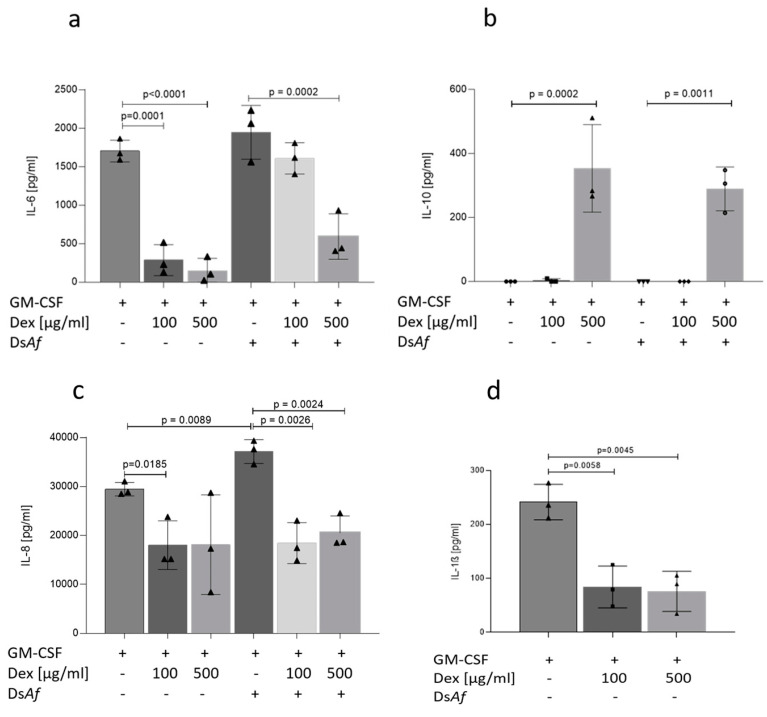
(**a**–**d**) Dexamethasone modifies cytokine levels towards anti-inflammatory in non- and *A. fumigatus* infected epithelial/immune lung tissues. Cell-free supernatants obtained from 24 h infected (**a**–**c**) versus uninfected (**a**–**d**), and 7 days Dex-treated versus untreated 3D lung models were analyzed for levels of secreted (**a**) IL-6, (**b**) IL-10, (**c**) IL-8, and (**d**) IL-1β using ELISA. Results are representative of three independent experiments, and concentrations were expressed as mean ± SD. Significance was calculated using an unpaired Student’s *t*-test. Signs (●, ▲) indicate numbers of independent repetition.

To determine whether dexamethasone altered cytokine profiles associated with fungal infections, basolateral supernatants were analyzed from *A. fumigatus*-infected NHBE-MDM cocultures and their uninfected counterparts after 24 h. A significant, dose-dependent decrease in IL-6 (Figure 4a), IL-8 (Figure 4c), and IL-1β (Figure 4d) and increase in IL-10 (Figure 4b) were analyzed in Dex-treated cultures as well as in Dex-treated and *A. fumigatus*-infected cocultures (Figure 4a–c). Thus, macrophage/epithelial cocultures exert an anti-inflammatory milieu also in the presence of *A. fumigatus,* when pre-treated with Dexamethasone over a 1-week period.

### 3.6. Dexamethasone Enhances Intra-Epithelial Fungal Invasion within the 3D Lung Model

**Figure 5:**
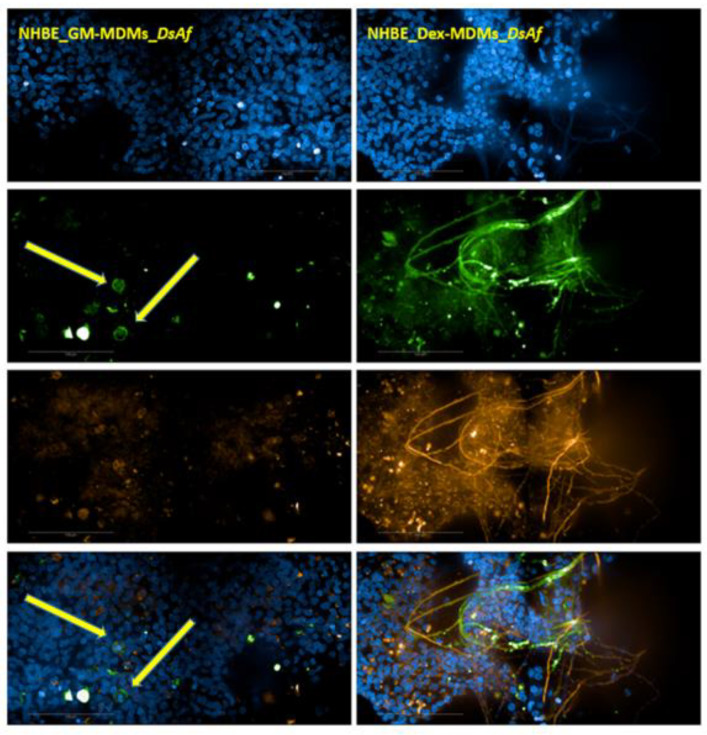
Growth and germination of DsRed-expressing *A. fumigatus* were enhanced within the dexamethasone-treated 3D lung model cocultured with macrophages. Pseudostratified primary epithelia, 40 days in ALI, are depicted. Arrows show intact GM–MDMs, which prevented growth 24 h post-infection. 20XW Objective, N.A. 1.0.

Lastly, we evaluated fungal growth in macrophage/epithelial cocultures in the presence and absence of the corticosteroid dexamethasone. The ability of such cocultures to inhibit the transition of conidia into invasive hyphae was assessed at 24 h post-infection. In comparison to untreated controls, hyphae overgrowth, also referred to as trans-epithelial invasion, was clearly visible in dexamethasone-treated conditions confirming that the environment established was immunosuppressive, as seen in Figure 5. 2D NHBE fungal infection experiments were also done to exclude effects unrelated to the presence of MDMs, and we found extensive hyphal growth in untreated as well as Dexamethasone-treated NHBE only conditions after overnight infection. This indicates that M1 macrophages, found to lesser levels under dexamethasone regimen within epithelial/macrophage cocultures, play a major role in combating *A. fumigatus* infection, and this line of defense is hampered by skewing macrophages into an M2 phenotype also within 3D immune/epithelial models.

## 4. Discussion

The common immunosuppressant, dexamethasone, is known to be quite potent, with its effects being influenced by long-term treatment as well as increased dosages [2]. This feature is one that has been associated with glucocorticoids (GCs) in general [12]. Our findings demonstrate that the 3D respiratory/immune lung model is a reliable in vitro platform for predicting clinical responses to immunosuppressive medications at all levels (low, normal, and supra-physiological) before clinical trials or therapy. We found within our model that dexamethasone-induced cell death was not a factor associated with treatment of immune or epithelial cells, but their functions were significantly hampered. Recently, we described the effects on low to high dexamethasone concentrations on macrophages only and found that dexamethasone at a range of 100 ng/mL to 500 µg/mL was not toxic to the cells and induced M1 to M2 macrophage polarization. Concentrations above 500 µg/mL caused macrophage cell death, which was due to high concentrations of ethanol (>2.4%), used as a solvent or the immunosuppressant [15]. Since there is limited data on the optimal dosages to apply for immunosuppressive therapeutics, a quantitative assessment was performed in preliminary dose-response experiments to exclude detrimental effects on pseudostratified epithelial layers. These comprised a range of 100 µg/mL to 500 µg/mL, and the concentrations were also tested for immune cells to be effective [15]. Since higher dexamethasone concentrations were described to exert faster immunosuppressive effects [24], we, too, included primary macrophages and not immortalized cell lines in our system, and dexamethasone was only applied once at the beginning of epithelial/macrophage coculture with a described half-life of 36–72 h [25], we decided on using the supra-physiological dexamethasone concentrations for coculture experiments to better monitor effects induced.

Immunosuppression using dexamethasone within the 3D respiratory epithelial/immune model compromised pro-inflammatory cytokine secretion. The crucial biomarkers associated with fungal clearance, IL-6, IL-1β, and IL-8, were reduced in dexamethasone-treated conditions at concentrations of 100 µg/mL and 500 µg/mL dexamethasone dissolved within the basal medium. The pulmonary epithelium is a great source of these biomarkers, which are released via toll-like receptor (TLR) 2 [9,26]. We also observed increased IL-10 levels within our tissue model, a potent anti-inflammatory cytokine [2] speculated to be the main reason for hyphae overgrowth that we also illustrated at a later time point. IL-6, on the other hand, was shown to be greatly influenced by the presence of TNF-α [27]. Despite not analyzing this cytokine directly, similar trans repression results have been observed in macrophages by us and others [17]. Overall the cytokine pattern observed in this study using long-term dexamethasone treatment and concomitant fungal infection, namely lowering of IL-1β, IL-6, and IL–8 and an increase in IL-10, implicates a shift from Th1 towards Th2, a pattern associated with suppressing antifungal properties [9,28].

Hyphae formation was increased in Dex-treated conditions within the macrophage/epithelial coculture model, and this is associated with extensive fungal invasion, immune system activation, and high fungal burden in clinical settings [29]. We postulate that the difference in invasion within our system is the induction of the aforementioned immunocompromised environment. In an attempt to clear a pathogen from the host system, macrophages must migrate within the tissues [30] and must be able to create an inflammatory environment. Since they are crucial players when it comes to preventing respiratory diseases, macrophage chemotaxis was also analyzed [31]. Our group previously demonstrated that dexamethasone downregulated essential migration molecules αmβ2 integrins and CD14 in treated M2-like macrophages [15], and the chemotaxis assay performed here confirmed the previously observed phenomenon as well as the identity with certainty of the resultant phenotype. By interrupting macrophage migration, dexamethasone interferes with antigen processing and presentation, which implies that the phenomenon would lead to obstruction of macrophage recruitment into invaded sites [32]. GMCSF was selected as the chemoattractant because it is the most abundant cytokine in the respiratory mucosal lining, associated with M1 macrophage polarization, and crucial for attracting various immune cells into the sites of invasion [15,26,33]. The impediment of surface glycoproteins was not only limited to immune cells because we also observed a significant downregulation of mucins MUC5AC and MUC1 on epithelial cells following dexamethasone treatment. Suppression of mucus production following dexamethasone treatment was already described earlier in cell lines or isografted rat trachea [7,34,35], and this is in accordance with our results from a highly differentiated human respiratory model. However, tight junctions (TJs) function as a border within the plasma membrane for selective permeability and also reflect the extent of differentiation [22,36]. This selective permeability is reported to affect calcium flux, therefore, directly impacting ciliary beat frequency and mucociliary clearance [37]. SAE barrier functions were clearly more sensitive to dexamethasone as observed through TEER measurements reaffirming the fact that NHBE cells play a superior role when it comes to preventing *A. fumigatus* growth within the respiratory system. NHBE cells did not experience any obvious stress-induced structural integrity damage due to dexamethasone treatment as assessed by measuring TEER. Previous reports describe the abilities of glucocorticoids to tighten cellular contacts in various epithelial cells in a TNF-α dependent blockage mechanism [22]. *A. fumigatus* binds and is internalized by lung cells [38], but enhanced barrier functions prevent its invasion capabilities [36]. In this study, infection of the GC-treated in vitro lung model revealed reduced mucus production (MUC 1 and MUC5AC) as well as suppressed ciliary bead frequencies. CBF is essential for the mechanical movement of mucus out of the lungs through the pharynx resulting in mucociliary clearance [5], which further confirms the increased hyphal growth seen in our epithelial/immune respiratory human model.

## 5. Conclusions

In conclusion, in vitro 3D lung models have proved to be useful platforms to simulate both immune-competent and -suppressed (both M1 and M2-inflammatory phases) pulmonary systems. Our study provides evidence that the platform consisting of highly differentiated NHBE cells grown in ALI under static conditions is reliable in assessing immunosuppressive pharmacologic agents within a physiologically relevant model. The immunosuppressed environment induced within this study was manifested by reduced mucus production, impeded CBF, reduced inflammatory cytokine secretion, increased CD163 expression, reduced macrophage migration, and increased fungal growth within the lung model. Therefore, the creation of a suppressive environment on both epithelial as well as immune side by long-term dexamethasone treatment favors the growth and invasion of pathogenic fungi. 

## Figures and Tables

**Table 1:** Stains used for Confocal Imaging and Flow Cytometry.

Antibody/Stain	Source	Cat Number	Concentration Applied
Alexa Fluor® 488 anti -human CD40	Biolegend, San Diego, CA	334318	40 µg/mL
Alexa Fluor® 488 Wheat Germ Agglutinin	Biotium, Hayward, CA	29022-1	5 µg/mL
Anti-Pan Cytokeratin	Invitrogen, Carlsbad, CA	53-9003-82	0.5 mg/mL
Alexa Fluor® 647 anti -human CD163	Biolegend, San Diego, CA	326508	10 µg/mL
Hoechst 33342	Sigma, St. Louis, MO	B1155	2 µg/mL
Mitotracker® Orange	Invitrogen, Carlsbad, CA	M7511	100–500 nM
Mouse anti-E-cadherin Alexa Fluor® 647	BD Pharmingen	560062	2.5 µg/mL
Mouse anti-Occludin Alexa Fluor® 594	Invitrogen, Carlsbad, CA	331594	5 µg/mL
MUC 1 488	Abcam, Cambdridge, UK	Ab196443	0.5 mg/mL
MUC 5 AC 555	Abcam, Cambdridge, UK	Ab218714	0.5 mg/mL
PE anti-human PKM2 (D78A4)	Cell Signaling Technology, Danvers, MA	983675	2.5 µg/mL

## Data Availability

All data from the study are presented in the figures or supplementary figures.

## Supplementary Materials

The following are available online at https://www.mdpi.com/2309-608X/7/3/221/s1. Figure S1: Clear phenotypic differences between dexamethasone-treated vs untreated NHBE in the unstained 3D Lung mode, Figure S2: CD40 and CD163 expression overview image, Figure S3: Sequential optical sections (Z-stacks) of M1 macrophage; Video S1: TJ integrity, Video S2a: control, Video S2b: dex 100ug, Video S3a: control, Video S3b: dex 100ug, Video S3c: dex 500ug, Video S4a-1: control, Video S4a-2: control, Video S4b-1: dex 100ug, Video 4b-2: dex 100ug, Video S4c-1: dex 500ug Video S4c-2: dex 500ug.
