# Supplementary material for: Dexamethasone Creates a Suppressive Microenvironment and Promotes Aspergillus fumigatus Invasion in a Human 3D Epithelial/Immune Respiratory Model
Source: J Fungi (Basel). 2021 Mar 18;7(3):221. doi: 10.3390/jof7030221 (PMC8003030; doi:10.3390/jof7030221)
Supplement: Supplementary file 1 [file jof-07-00221-s001.zip › 1-jof-1152227-supple-vidio+ms/3-jof-1152227-supple.docx]

**Supplementary Figures and Figure Legends**

**Supplementary Figure S1.** Clear phenotypic differences between dexamethasone-treated vs untreated NHBE in the unstained 3D Lung model, Brightfield imaging using Operetta CLS High Content Analysis system (Perkin Elmer), 42 days in ALI X5air objective, N. A 0.16 and X20W Objective N.A. 1.0

**Supplementary Figure S2**. CD40 and CD163 expression overview image. Dexamethasone (100ug/ml) treated macrophages expressed both CD40 (Alexa Fluor® 488, green) and CD163 (Alexa Fluor® 647, red) surface markers within 3D lung model indicating the establishment of an immunosuppressed micro-environment, NHBE Passage 2, 100,000 cells per TW, 82 days in ALI. Scale bar_ 200um

**Supplementary Figure S3**. Sequential optical sections (Z-stacks) of M1 macrophage Chemotaxis within the 3D lung model depicting serially thin 0.5 – 1.5um optical sections of the co - culture membrane with CD40^+^ untreated macrophages in the basolateral side while CD163^+^ macrophages largely remain apical. Z - stacks were collected during experimentation in order to reflect the depth of the membrane as well as reveal any internal variations in auto fluorescence emission.
